# Oxidation of β2-glycoprotein I associates with IgG antibodies to domain I in patients with antiphospholipid syndrome

**DOI:** 10.1371/journal.pone.0186513

**Published:** 2017-10-19

**Authors:** Maria Gabriella Raimondo, Charis Pericleous, Anna Radziszewska, Maria Orietta Borghi, Silvia Pierangeli, Pier Luigi Meroni, Ian Giles, Anisur Rahman, Yiannis Ioannou

**Affiliations:** 1 Centre for Rheumatology Research, UCL Division of Medicine, London, United Kingdom; 2 Department of Clinical Sciences and Community Health, University of Milan, Milan, Italy; 3 Arthritis Research UK Centre for Adolescent Rheumatology, UCL Division of Medicine, London, United Kingdom; 4 IRCCS Istituto Auxologico Italiano, Milan, Italy; 5 Division of Rheumatology, Department of Medicine, University of Texas Medical Branch, Galveston, Texas; Institut d'Investigacions Biomediques de Barcelona, SPAIN

## Abstract

Domain I (DI) of beta-2-glycoprotein I (β_2_GPI) contains the immunodominant epitope for pathogenic antiphospholipid antibodies (aPL). DI is exposed in the linear form of the molecule but not in the circular form that comprises 90% of serum β_2_GPI. The majority of circulating β_2_GPI is biochemically reduced with two free thiols in Domain V. However, increased levels of oxidised β_2_GPI are found in patients with antiphospholipid syndrome (APS). It is not known whether oxidation of β_2_GPI favours the linear form of the molecule and thus promotes development of anti-DI antibodies. We investigated whether the proportion of oxidised β_2_GPI associates with the presence of anti-DI in APS patients.

Serum samples from 44 APS patients were screened for IgG, IgM and IgA anti-DI, anti-β_2_GPI, anti-cardiolipin (anti-CL) and biochemically reduced β_2_GPI. A negative correlation was found between the proportion of β_2_GPI in the biochemically reduced form and IgG anti-DI levels (r = -0.54, p = 0.0002), but not with IgM or IgA anti-DI. Moreover, the proportion of β_2_GPI in the reduced form was lower in IgG anti-DI positive than anti-DI negative APS patients (p = 0.02). The relative amount of reduced β_2_GPI was no different between patients who were positive or negative for IgG, IgM and IgA anti-β_2_GPI or anti-CL. This study demonstrates that oxidised β_2_GPI lacking free cysteine-thiol groups most closely associates with IgG anti-DI positivity compared to IgG anti-CL and anti-β_2_GPI. Future studies are required to ascertain the directionality of this association to define causation.

## Introduction

Antiphospholipid syndrome (APS) is a systemic autoimmune disease characterized by vascular thrombosis and recurrent pregnancy morbidity in the presence of serum antiphospholipid antibodies (aPL) [1]. Beta-2-glycoprotein I (β_2_GPI—serum concentration 50–400 μg/mL) is the main antigenic target of aPL in patients with APS [2]. β_2_GPI has five homologous domains (DI-V) and the N-terminal domain I (DI) contains the immunodominant epitope [3]. *In vivo* and *in vitro* studies showed that IgG antibodies targeting β_2_GPI-DI (anti-DI) represent a key pathogenic sub-population of anti-β_2_GPI and strongly associate with vascular thrombosis in patients with APS [4,5].

β_2_GPI *in vivo* exists in two forms [6]; the circular form, maintained by interaction between the first and fifth domain thus potentially hiding the epitope on DI, and the fishhook-like, linear shape in which the epitope is exposed [6]. β_2_GPI also exists in two interconvertible biochemical configurations. These are the reduced form, containing free thiols at cysteine 288 and cysteine 326 within domain V, and the oxidised form, in which these thiols form a disulphide bond [7,8]. In a large study of over 400 subjects, Ioannou et al [9] compared the levels of total and reduced β_2_GPI in patients with APS, healthy controls, autoimmune disease controls (with systemic lupus erythematosus or Sjogren’s syndrome but no APS) and patients who suffered thrombosis in the absence of APS. Compared to all the other groups, the patients with APS had significantly higher levels of total β_2_GPI but significantly lower proportion of reduced β_2_GPI. Thus, patients with APS have higher levels of oxidised β_2_GPI than other groups. Furthermore, Giannakopoulos and Krilis showed that affinity-purified antiphospholipid antibodies from patients with APS bound more strongly to oxidised than to reduced β_2_GPI [10].

It is not known, however, whether the circular form of β_2_GPI is primarily reduced and whether oxidation of β_2_GPI favours linearization of the protein with exposure of antigenic epitopes on DI. Only structural or biophysical studies could answer this question. Binding studies, however, can be used to test the hypothesis that the proportion of oxidised β_2_GPI in serum of patients with APS correlates with the level of anti-DI antibodies. This was the purpose of the study reported here.

## Patients and methods

### Patient samples

Serum samples were collected from 44 patients from three centres (University of Milan [Milan, Italy], University College London [London, UK], University of Texas Medical Branch [Galveston, US]). All 44 patients fulfilled the revised classification criteria for APS [11] (38 primary APS [PAPS] and 6 systemic lupus erythematosus-related APS [SLE-related APS]). All 6 patients with SLE fulfilled the American College of Rheumatology revised classification criteria [12].

### Ethics approval

The study was approved by the National Research Ethics Committee London-Hampstead (Reference 12/LO/0373). Patients provided written consent to provide samples for the study.

### ELISA for quantifying the total amount of β_2_GPI

A capture assay for quantifying total β_2_GPI levels within human serum was performed as previously [9], with some modifications. The capture antibody used was a monoclonal mouse anti-human β_2_GPI antibody (6C4C10 1:33000 dilution, kind gift from Dr M Iverson, La Jolla Pharmaceuticals) and the detecting antibody was goat polyclonal anti-human β_2_GPI antibody (Thermo Scientific). Serial dilutions of an in-house standard consisting of pooled serum from 10 healthy controls (HC) was used to construct a standard curve for the ELISA. The concentration of β_2_GPI in the pooled-serum in-house standard was validated with a commercial kit for β_2_GPI quantification (Abcam). Therefore we were able to calculate the absolute concentration of β_2_GPI in each sample in micrograms per ml.

### ELISA for quantifying the reduced proportion of β_2_GPI

Measurement of the amount of β_2_GPI that is reduced is based on labelling of free thiols of β_2_GPI as described previously [7,9], using the biotin-conjugated agent N-(3-maleimidylpropionyl) biocytin (MPB). Patient serum (50 microlitres) was incubated with 4mM MPB at room temperature in the dark for 30 minutes with agitation, then diluted 50-fold in 20mM HEPES buffer (pH 7.4) and incubated for a further 10 minutes in the dark. Unbound MPB was removed by acetone precipitation and the protein pellet was re-suspended in PBS-0.05% Tween, to a final dilution of 4000-fold. These samples now contained MPB bound to all proteins that had previously carried free thiols and were coated on streptavidin plates to capture the MPB-labelled proteins. The plates were blocked with 2% bovine serum albumin (BSA) in phosphate buffered saline (PBS) containing 0.1% Tween and washed three times with PBS-0.1% Tween. In order to detect only β_2_GPI labelled with MPB (and not other MPB-labelled proteins) we then added a specific murine anti-β_2_GPI monoclonal antibody (4B2E7) at a concentration of 25nM and incubated for one hour at room temperature. After washing again with PBS-0.1% Tween, alkaline phosphatase conjugated goat anti-mouse IgG was added for one hour at room temperature and the optical density was read at 405nm after addition of chromogenic substrate. As a positive control on each plate we used an in-house standard, which was the same pool of serum from 10 HC used in the total β_2_GPI assay, but on this occasion treated with MPB to label free thiols, as above.

**Fig 1:**
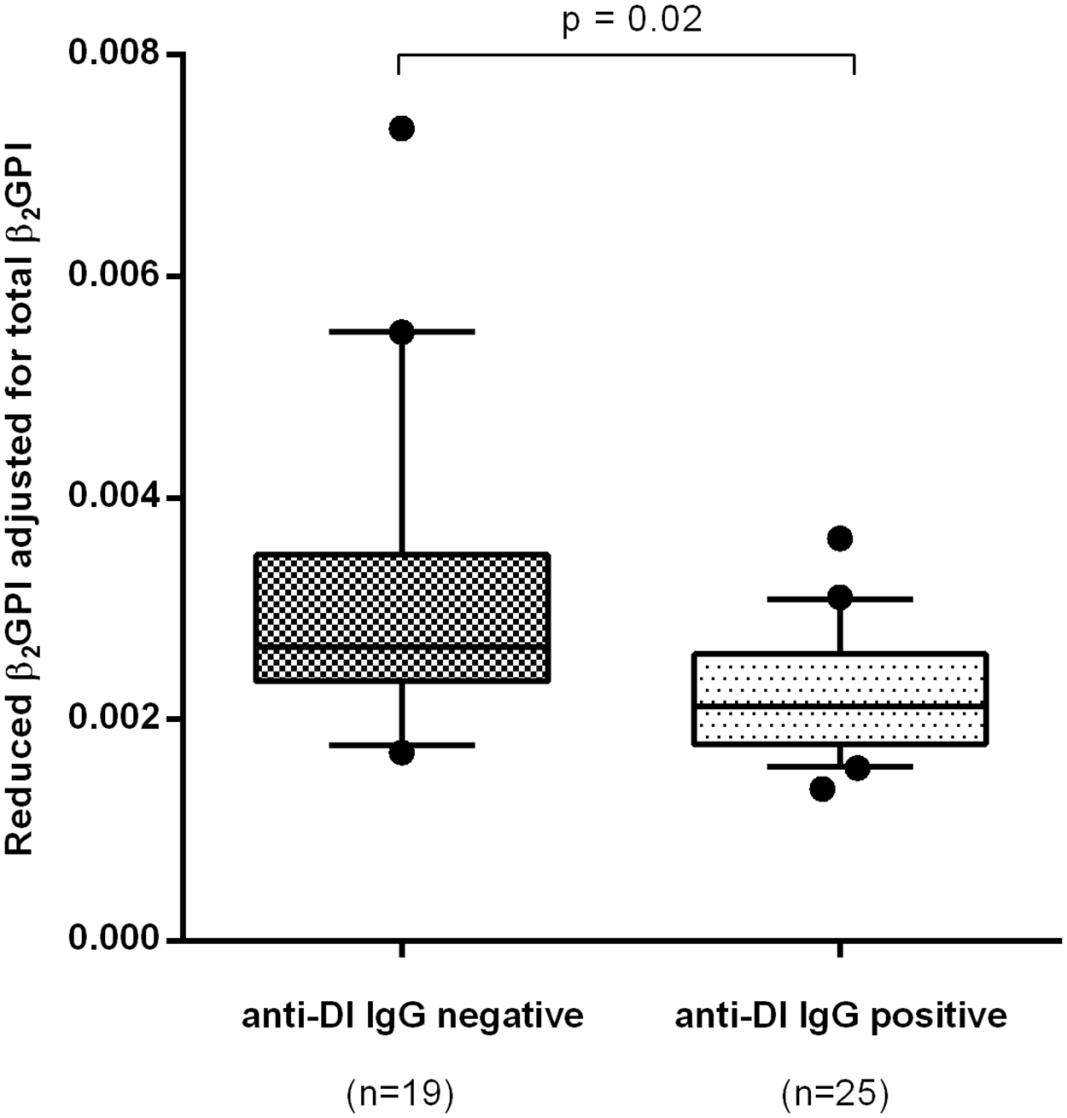
Association of IgG anti-DI-positivity with an elevated proportion of circulating oxidised β_2_GPI in APS patients. Levels of reduced β_2_GPI were adjusted for the total amount of β_2_GPI for each sample. Anti-DI positivity was based on a cut-off established as the 99th percentile of 200 HC. Samples from APS patients who were positive for anti-DI had a significantly lower proportion of reduced β_2_GPI as compared to those from anti-DI negative patients.

The result of the assay was expressed as a ratio of OD of sample/OD of positive control. This gave a measure of the amount of reduced β_2_GPI in each sample, which was then divided by the total β_2_GPI concentration (derived as in the previous section). Adjustment for total β_2_GPI is necessary in order that the results of the assay should give a measure of the proportion of β_2_GPI which is reduced in each sample. This value–“reduced β_2_GPI adjusted for total β_2_GPI” is shown on the y-axis of Fig 1.

### ELISA for measuring levels of aPL antibodies

Patient samples were tested for IgG, IgM and IgA anti-DI, anti-β_2_GPI and anti-cardiolipin (anti-CL) as described previously [3,13,14]. We classified each sample as being positive or negative for each of these antibodies, based on a cut-off of the 99^th^ centile of 200 HC previously tested [3,13,14]. Historical lupus anticoagulant (LA) activity was recorded from the medical records of the patients.

### Statistics

Non-parametric Spearman's correlation and Mann-Whitney U test were applied for the statistical analyses.

## Results

### Clinical and serological profile of the patients tested

The clinical, demographic and serological features of the patients are shown in Table 1. The APS group involved all clinical sub-types of APS including patients with a history of vascular thrombosis alone, pregnancy morbidity alone or both these outcomes. Importantly, we included sufficient positive and negative cases for each of the 9 antibodies tested (i.e. IgG, IgA and IgM to each of CL, β_2_GPI and DI) to make comparisons between antibody-positive and negative groups meaningful. Ninety percent of subjects were positive for LA.

**Table 1:** Clinical features and serology of APS patients.

	Patients (n = 44)
**Median age (SD)**	42 yr (10.6)
**Sex (F:M)**	35:9
**Ethnicity (%)**	Caucasian (97.7)
**Diagnosis (PAPS:SAPS)**	38:6
**VT+/PM+ (%)**	9 (20.5)
**PM+ (%)**	14 (31.8)
**VT+ (%)**	21 (47.7)
**IgG anti-DI positive**	56.8%
**IgM anti-DI positive**	38.6%
**IgA anti-DI positive**	47.7%
**IgG anti-β**_**2**_**GPI positive**	72.7%
**IgM anti-β**_**2**_**GPI positive**	25%
**IgA anti-β**_**2**_**GPI positive**	54.5%
**IgG anti-CL positive**	77.2%
**IgM anti-CL positive**	15.9%
**IgA anti-CL positive**	59%
**LA activity**	90%

(PAPS: primary antiphospholipid syndrome; SAPS: secondary antiphospholipid syndrome; VT: vascular thrombosis; PM: pregnancy morbidities).

### Biochemically reduced β_2_GPI is negatively associated with IgG anti-DI but not with other isotypes of anti DI nor with IgG anti-β_2_GPI

A significant negative correlation was obtained between the proportion of β_2_GPI in the reduced form and IgG anti-DI levels (r = -0.54, p = 0.0002), but not with IgM or IgA anti-DI (r = 0.08, p = 0.59 and r = -0.19, p = 0.21, respectively). To investigate this finding further, we stratified the patients according to positivity or negativity for IgG anti-DI and a significant difference in proportion of reduced β_2_GPI was found between the two groups (Fig 1). Although anti-DI antibodies represent a sub-population of anti-β_2_GPI, no significant correlation was found between the reduced proportion of β_2_GPI and IgG, IgM or IgA anti-β_2_GPI (r = -0.17, p = 0.25; r = -0.09, p = 0.56; r = -0.01, p = 0.94 respectively) and no significant difference existed dividing the samples according to IgG anti-β_2_GPI positivity (p = 0.89). A similar analysis was then performed with IgG, IgM and IgA anti-CL levels. A significant association was obtained between the reduced proportion of β_2_GPI and IgG anti-CL titre (r = -0.34, p = 0.02) though this association was weaker than that seen for IgG anti-DI. However, the proportion of β_2_GPI in the reduced form did not show any difference between IgG anti-CL positive and negative groups (p = 0.06). No correlation was found between the reduced proportion of β_2_GPI and either IgM or IgA anti-CL titre (r = -0.11, p = 0.46, r = -0.15, p = 0.31, respectively). Finally, LA positivity did not associate with proportion of β_2_GPI in its reduced form in APS patients (p = 0.07).

## Discussion

In this study we report, for the first time, a statistically significant negative correlation between levels of IgG anti-DI antibodies and proportion of β_2_GPI in the reduced form in patients with APS.

There are two possible biological mechanisms explaining this finding and we believe that both may be important in patients with APS. One possibility is that the oxidised protein is more likely than the reduced form to be linear, with the DI epitope exposed, thus stimulating the formation of anti-DI antibodies. It is known from crystallographic studies that the linear form of purified β_2_GPI does not contain any free thiols [15] and the protein is oxidised. Ioannou *et al* showed that at least 46% of circulating β_2_GPI is in the reduced form [9]. As the labelling of free thiols with MPB is not 100% efficient, this figure is likely to be an under-estimate. Using analysis of trypsin digestion fragments of both forms of β_2_GPI, Agar *et al* [6] showed that Lysine 305 and Lysine 317 in DV were not accessible to trypsin in the circular form but were accessible in the linear form. This finding suggests that, in the circular form, DI interacts with DV at a site lying between Cysteine 288 and Cysteine 326, which would make formation of a disulphide bond between those residues difficult and would favour reduction rather than oxidation. Direct proof however, of any link between the reduced form and the circular form requires further structural studies.

An alternative explanation is that, rather than oxidised β_2_GPI triggering formation of anti-DI antibodies, it is the anti-DI antibodies that stabilise the oxidised form, thus pushing the redox equilibrium of β_2_GPI in the direction of oxidation. If the linear form of β_2_GPI does indeed favour oxidation, as described above, binding of anti-DI antibodies to whole linear β_2_GPI could prevent DI from binding to DV and forming a circular structure, stabilising the linear form of the molecule and thus favouring oxidation. Agar *et al* [6] demonstrated that linearization of β_2_GPI is observed in the presence of aPL which also supports this theory. Both these hypotheses, regarding the antigenic drive for anti-DI production and the effect of these antibodies on the redox state of β_2_GPI, require further investigation.

The proportion of reduced β_2_GPI did not correlate with levels of antibodies to whole β_2_GPI. The most likely reason for this is that anti-DI antibodies constitute only a subgroup of anti-β_2_GPI antibodies and that antibodies to other domains of β_2_GPI can be triggered equally well by reduced or oxidised β_2_GPI. Furthermore, there is no reason to suppose that antibodies to Domains II-IV of β_2_GPI would stabilise the linear rather than the circular form of the molecule.

IgM anti-β_2_GPI and anti-CL are included in the classification criteria for APS [11] and recent studies suggest that IgA might be pathogenic as well [16]. However, we did not show a correlation between IgM or IgA anti-DI (or anti-β_2_GPI or anti-CL) antibodies and the proportion of reduced β_2_GPI. The difference between results obtained for the IgM and IgG isotypes of anti-DI may be linked to the fact that class switching from IgM to IgG is associated with affinity maturation of antibodies in the presence of antigen. Thus the presence of oxidised β_2_GPI may be important at this stage of development of an autoreactive B cell producing anti-DI antibodies, leading to production of high affinity IgG anti-DI rather than lower affinity IgM anti-DI. Equally, higher affinity IgG anti-DI may stabilise linear β_2_GPI more effectively than IgM.

The difference between the results for IgA and IgG anti-DI could arise from the fact that circulating IgA levels are much lower than those of IgG so that any effect of anti-DI antibodies in stabilising oxidised β_2_GPI would be seen primarily for IgG. Differences in properties between IgA and IgG antibodies to the same antigen have been noted in other autoimmune diseases. For example, a recent study in patients with rheumatoid arthritis showed that IgG anti-cyclic citrullinated peptide (CCP) antibodies were associated with the shared epitope whereas IgA anti-CCP were not [17]. Conversely, in the same study, only IgA but not IgG anti-CCP were associated with smoking.

In conclusion, we have shown a specific association between IgG anti-DI antibodies and increased oxidation of β_2_GPI in patients with APS, which is not seen with other isotypes of anti-DI or with anti-CL or anti-β_2_GPI antibodies. Further studies to look at ability of monoclonal or polyclonal anti-DI antibodies to modify the redox equilibrium of β_2_GPI may be interesting. It would be hard to carry out experiments comparing immunogenicity of oxidised versus reduced β_2_GPI in animal models because it is difficult to maintain purified β_2_GPI in its reduced form *in-vitro*. However, future protein structural studies confirming that reduced β_2_GPI relates to the circular form would aid future studies to define the causal relationship between the redox state of β_2_GPI and anti-DI antibodies.
